# OsIRO3 Plays an Essential Role in Iron Deficiency Responses and Regulates Iron Homeostasis in Rice

**DOI:** 10.3390/plants9091095

**Published:** 2020-08-25

**Authors:** Wujian Wang, Jun Ye, Yanran Ma, Ting Wang, Huixia Shou, Luqing Zheng

**Affiliations:** 1 College of Life Sciences, Nanjing Agricultural University, Nanjing 210095, China; 2016216008@njau.edu.cn (W.W.); 2018116033@njau.edu.cn (J.Y.); 2017116045@njau.edu.cn (Y.M.); 2018816154@njau.edu.cn (T.W.); 2 State Key Laboratory of Plant Physiology and Biochemistry, College of Life Sciences, Zhejiang University, Hangzhou 310058, China; huixia@zju.edu.cn

**Keywords:** rice, iron, Fe deficiency, transcription factor, OsIRO3, OsNAS3, nicotianamine

## Abstract

Iron (Fe) homeostasis is essential for plant growth and development, and it is strictly regulated by a group of transcriptional factors. Iron-related transcription factor 3 (OsIRO3) was previously identified as a negative regulator for Fe deficiency response in rice. However, the molecular mechanisms by which OsIRO3 regulate Fe homeostasis is unclear. Here, we report that OsIRO3 is essential for responding to Fe deficiency and maintaining Fe homeostasis in rice. OsIRO3 is expressed in the roots, leaves, and base nodes, with a higher level in leaf blades at the vegetative growth stage. Knockout of *OsIRO3* resulted in a hypersensitivity to Fe deficiency, with severe necrosis on young leaves and defective root development. The *iro3* mutants accumulated higher levels of Fe in the shoot under Fe-deficient conditions, associated with upregulating the expression of *OsNAS3*, which lead to increased accumulation of nicotianamine (NA) in the roots. Further analysis indicated that OsIRO3 can directly bind to the E-box in the promoter of *OsNAS3*. Moreover, the expression of typical Fe-related genes was significantly up-regulated in *iro3* mutants under Fe-sufficient conditions. Thus, we conclude that OsIRO3 plays a key role in responding to Fe deficiency and regulates NA levels by directly, negatively regulating the *OsNAS3* expression.

## 1. Introduction

Iron (Fe) is an indispensable micronutrient for plants and animals. It acts as a cofactor for a number of enzymes and plays an essential role in many metabolic processes [1]. Fe deficiency is one of the most prevalent nutrient deficiencies in the world; it affects more than one third of the global population [2]. Plants, which are the major Fe sources for humans, take up inorganic Fe from the soil. Although Fe is abundantly present in the earth’s crust, its bioavailability is very low due to the insolubility of inorganic Fe, especially in calcareous soils, which account for about 30% of the world’s cultivated soils [3]. Therefore, disclosing the mechanism underlying Fe homeostasis in plants is important to human health.

Higher plants use two major Fe uptake strategies under low-Fe conditions: Strategy I and Strategy II [3,4]. Non-grass plants, such as *Arabidopsis*, employ Strategy I, which mainly comprises three processes: (1) roots secrete protons into the rhizosphere that lower the pH by increasing the activity of the H^+^-ATPase 2(AHA2), resulting in higher solubility of the ferric (Fe^3+^) form; (2) Fe^3+^ is reduced to the ferrous (Fe^2+^) form by the plasma membrane protein ferric reduction oxidase 2 (FRO2); and (3) Fe^2+^ is taken up by iron-related transporter 1 (IRT1) [3,5,6,7]. In contrast, grass plants that use Strategy II and secrete mugineic acid family phytosiderophores (MAs) through transporter of mugineic acid 1 (TOM1) to bind to Fe^3+^, and then Fe^3+^-MA complexes are taken up into roots by yellow stripe 1 (YS1) or YS1-like (YSL) proteins [8,9,10,11].

To adapt to fluctuating environments, plants have a set of sophisticated regulatory systems at transcriptional and post-transcriptional levels. In *Arabidopsis*, the basic helix-loop-helix (bHLH) fer-like iron deficiency-induced transcription factor (FIT) forms hetero-dimers with subgroup Ib bHLH proteins AtbHLH38, AtbHLH39, AtbHLH100, and AtbHLH101, and these dimers positively regulate the major Strategy I-type Fe acquisition genes, including *IRT1*, *FRO2,* and *AHA2* [12,13,14]. In addition to the FIT regulator network, the PYE (popeye) network also participates in Fe homeostasis [15]. Under Fe deficiency conditions, *PYE* is strongly induced in the pericycle. The *pye* mutant is sensitive to various low Fe growth conditions compared with wild type (WT). PYE can directly and negatively regulate the expression of three genes *ferric reduction oxidase 3* (*FRO3*), *nicotainamine synthase 4* (*NAS4*), and *zinc-induced facilitator 1* (*ZIF1*) that are involved in Fe homeostasis [16]. PYE can interact with some subgroup IVc bHLH transcription factors (bHLH034/104/105/115). These bHLH transcription factors have been demonstrated to regulate Fe homeostasis by binding to the E-box in the promoter of bHLH38/39/100/101 and PYE [17,18,19,20]. Clade IVb bHLHs comprise three bHLHs (bHLH11, bHLH121, and PYE). bHLH11 negatively regulates FIT-dependent Fe uptake and modulates Fe levels in *Arabidopsis* [21]. bHLH121 functions as a master positive regulator of Fe homeostasis; it acts upstream of FIT in concert with ILR3 and its closest homologs [22,23,24]. These results suggest a number of bHLHs are critical for modulating Fe homeostasis in *Arabidopsis*.

In rice, a number of bHLH transcription factors have also been identified to regulate Fe homeostasis. Iron-related transcription factor 2 (OsIRO2), a homologue of *Arabidopsis* bHLH38/39/100/101, plays a critical role in Fe deficiency responses by positively regulating Strategy II-associated genes [25,26]. In rice, OsbHLH156, a homologue of FIT, interacts with OsIRO2 and positively regulates Strategy II Fe acquisition through localizing of OsIRO2 into the nucleus [27,28]. By contrast, plants that overexpress *OsIRO3* are sensitive to Fe deficiency, and both Strategy I- and Strategy II-associated genes are suppressed in these plants, suggesting IRO3 is a negative regulator of Fe deficiency responses in rice [29]. Amino acid sequence analysis showed that OsIRO3 is a homology protein of PYE, suggesting the regulatory mechanism of PYE in *Arabidopsis* and OsIRO3 in rice was conserved. The expression of *OsIRO2*/*OsIRO3* is regulated by the subgroup IVc bHLH transcription factor positive regulators of iron homeostasis (OsPRI1/OsbHLH060, OsPRI2/OsbHLH058, OsPRI3/OsbHLH059) [30,31,32]. Rice proteins OsPRI1/2/3 are homologies of bHLH034/104/105/115, which are positive regulators of iron homeostasis in *Arabidopsis* [29,30,31,32]. Considering that *OsIRO2* is also a homology gene of bHLH038/039/100/101, the positive regulation network of Fe homeostasis between rice and *Arabidopsis* is also conserved*. OsbHLH133* is induced by Fe-deficient conditions in rice, and it is an essential regulator of proper Fe distribution between roots and shoots [33].

OsIRO3 has been reported as a negative regulator of the Fe deficiency response in rice, mainly based on the phenotype and gene expression analysis gained from *OsIRO3* overexpression lines [29]. However, the exact regulatory mechanism of OsIRO3 in Fe deficiency responses is still unclear, and the direct downstream genes of OsIRO3 are also unknown. In this study, we analyzed phenotypes of *OsIRO3*-knockout mutants under both Fe-deficient and Fe-sufficient conditions. Functional analysis revealed that in rice, OsIRO3 played an essential role for the response to Fe deficiency and for maintaining Fe homeostasis. Importantly, OsIRO3 can directly bind to the promoter of *OsNAS3* and negatively regulate its expression.

## 2. Results

### 2.1. Expression Analysis of OsIRO3

**Figure 1:**
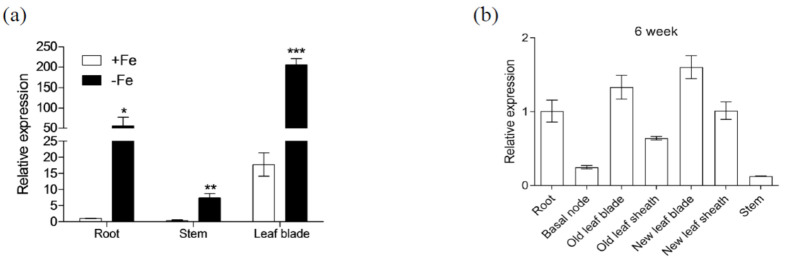
Expression pattern of *OsIRO3.* (**a**) Relative expression of *OsIRO3* in root, stem, and leaf blade under both Fe-sufficient conditions (+Fe) or Fe-deficient conditions (−Fe). Two-week-old plants were transferred to +Fe or −Fe for 7 d. The root, leaf blades, and stems were sampled for expression analysis. Asterisks above bars indicate significant differences (* *p* < 0.05, ** *p* < 0.01, *** *p* < 0.001) compared with the Fe-sufficient condition (+Fe), as determined by two-tailed Student’s *t* test. (**b**) Relative expression of *OsIRO3* in different organs at vegetative growth stage. Different tissues of 6–week-old rice grown in solution were sampled for expression analysis. The expression level relative to the expression in +Fe root (**a**) or root (**b**) was shown. Data are given as the means ± SD of three biological replicates.

We performed quantitative real-time polymerase chain reaction (qPCR) analysis to investigate the expression pattern of *OsIRO3*. The expression levels of *OsIRO3* in roots, stems, and shoots were significantly upregulated by Fe deficiency (Figure 1a). The transcript abundance of *OsIRO3* was higher in leaf blades than in roots and stems under both Fe-sufficient and Fe-deficient conditions (Figure 1a). To investigate the tissue-specific *OsIRO3* expression pattern, we examined its levels in different tissues from 6-week-old rice plants. *OsIRO3* was highly expressed in roots, leaf blades, and leaf sheaths; it was also expressed in the stems and basal nodes (Figure 1b).

### 2.2. Knockout of OsIRO3 Leads to Hypersensitivity to Fe Deficiency

**Figure 2:**
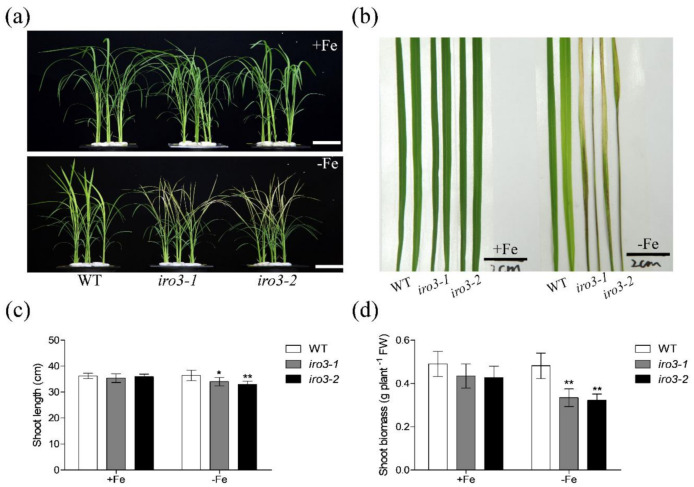
Shoot phenotype analysis of *iro3* mutants. (**a**) Shoot growth performance of wild type (WT) and *iro3* mutants under +Fe or −Fe conditions. (**b**) The two newly developed leaves of WT and *iro3* mutants under +Fe or −Fe conditions. (**c**) Shoot length of WT and *iro3* mutants. (**d**) Shoot biomass of WT and *iro3* mutants. Fourteen-day-old seedlings were transferred to Fe-deficient conditions (−Fe) or Fe-sufficient conditions (+Fe) for 8 d. The *iro3-1* mutant contains an insertion of ‘A’; the *iro3-2* mutant contains an insertion of ‘T’. Data are given as the means ± SD of six biological replicates. All data were compared with the WT. Asterisks indicate significant differences of WT and *iro3* mutants based on two-tailed Student’s *t* test (* *p* < 0.05, ** *p* < 0.01). Bars = 5 cm in (**a**) and 2 cm in (**b**).

**Figure 3:**
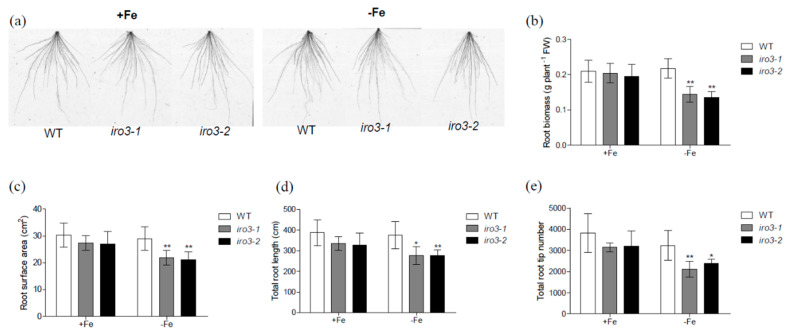
Root phenotype analysis of *iro3* mutants. (**a**) Root growth performance of WT and *iro3* mutants grown under +Fe or −Fe conditions. (**b**–**e**) Root parameters of WT and *iro3* mutants grown under +Fe or −Fe conditions. Root biomass (**b**), root surface area (**c**), total root length (**d**), and total root tip number (**e**) of WT and *iro3* mutants under +Fe and −Fe conditions. Fourteen-day-old seedlings were transferred to −Fe or +Fe for 8 d. The *iro3-1* mutant contains an insertion of ‘A’; the *iro3-2* mutant contains an insertion of ‘T’. Data are given as the means ± SD of six biological replicates. All data were compared with the WT. Asterisks indicate significant differences of WT and *iro3* mutants based on two-tailed Student’s *t* test (* *p* < 0.05, ** *p* < 0.01).

To further investigate the function of OsIRO3 in Fe homeostasis in rice, we used the CRISPR/Cas9 system to create loss-of-function mutants of *OsIRO3*. Two *OsIRO3* gene sequences in the first exon were selected as mutation sites and were used in two independent rice transformations, respectively (Figure S1a). The homozygous *iro3* mutants (i.e., *iro3-1* and *iro3-2*) were identified by sequencing. Both mutants were affected by a frame shift due to one base insertion (Figure S1b), which resulted in the OsIRO3 protein lacking the basic helix-loop-helix (bHLH) domain due to the premature termination codon appearing in the N-terminal of OsIRO3 (Figure S2). Then, we compared the growth capacity of WT and *OsIRO3*-knockout mutants (*iro3-1* and *iro3-2*) under both Fe-sufficient and Fe-deficient conditions (Figure S1). In the presence of Fe, the growth performances of the two *iro3* mutants were similar to that of WT plants (Figure 2 and Figure 3). These results indicate that OsIRO3 affects neither the normal growth nor the basal metabolism of rice. However, the new leaves of *iro3* mutants appeared severely necrotic after being grown under Fe-deficient conditions for 8 days, whereas WT leaves only showed chlorosis, which is typical of Fe deficiency (Figure 2a,b). The shoot height and fresh weight of both *iro3* mutants were significantly lower than those of the WT under Fe-deficient conditions (Figure 2c,d). The necrosis appeared in *iro3* mutant leaves might experience reactive oxygen species (ROS)-induced hypersensitive cell death. Under Fe-deficient conditions, we found the *iro3* mutants accumulated higher superoxide (O_2_^−^) and hydrogen peroxide (H_2_O_2_) levels by Nitro blue tetrazolium (NBT) and 3,3′–diaminobenzidine (DAB) staining, respectively (Figure S3). These results indicate that the shoots of *iro3* mutants are more sensitive to Fe deficiency than those of WT.

We also found alterations in root morphology of the *iro3* mutants in response to Fe deficiency compared with that of WT plants (Figure 3). There was significantly less root biomass in both *iro3* mutants compared with that of WT under Fe-deficient conditions (Figure 3a,b). Furthermore, measurement of various root indices revealed that *iro3* mutants had less root surface area and total root length and a lower total root tip number than those of WT plants under Fe-deficient conditions (Figure 3c–e). Similar to shoots, there was no difference between WT and the *iro3* mutants’ roots under Fe-sufficient conditions (Figure 3). These results suggest that knockout of *OsIRO3* leads to decreased tolerance of shoot and root responses to Fe deficiency. Hence, *iro3* mutants are hypersensitive to Fe deficiency, and OsIRO3 is required for rice survival under Fe deficiency stress.

### 2.3. Fe Concentration Analysis in WT and iro3 Mutants

**Figure 4:**
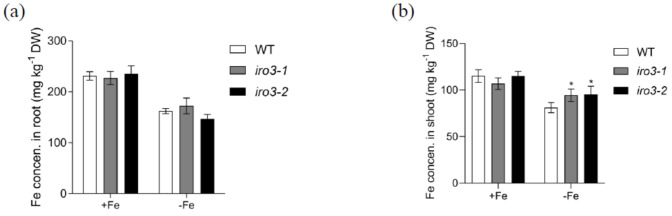
Fe concentration in WT and *iro3* mutants. (**a**) Root and (**b**) shoot Fe concentration of WT, *iro3-1*, and *iro3-2* under +Fe or −Fe conditions. Fourteen-day-old seedlings of WT, *iro3-1,* and *iro3-2* were transferred to nutrient solution containing 0 or 50 μM Fe (III)-EDTA and grown for 8 d. Root and shoot Fe content of WT, *iro3-1*, and *iro3-2* were analyzed. Data are given as the means ± SD of four biological replicates. All data were compared with the WT. Significant differences from the mutant and WT are indicated by * *p* < 0.05, as determined by two-tailed Student’s *t* test.

Next, to investigate whether knockout of *OsIRO3* affects Fe uptake and transport in rice plants, we measured Fe concentration in WT and *iro3* mutants under both Fe-sufficient and Fe-deficient conditions. Under Fe-sufficient conditions, there was no difference in root or shoot Fe concentration between WT and *iro3* mutants (Figure 4). By contrast, under Fe-deficient conditions, while there was no difference in the roots, the Fe concentration was significantly higher in the shoots of mutant compared with those of WT plants (Figure 4a,b). These results suggest that OsIRO3 is involved in maintaining Fe accumulation in shoots under Fe-deficient conditions.

### 2.4. Expression of Fe Homeostasis Genes in Roots of WT and iro3 Mutants

**Figure 5:**
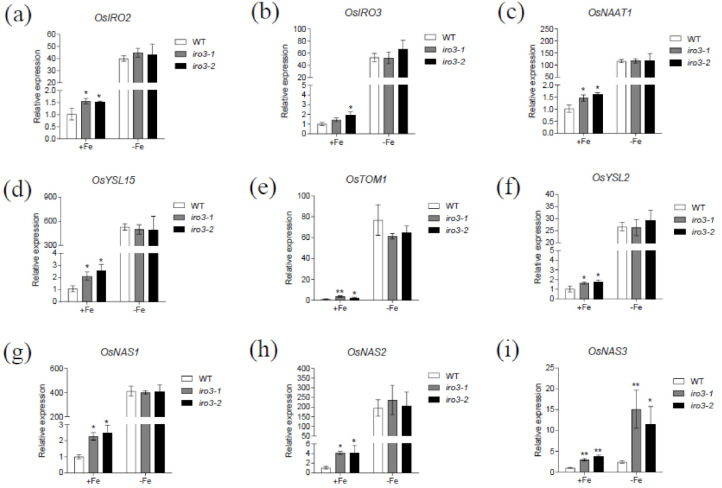
Expression of Fe deficiency responsive genes in the *iro3* mutants. Fourteen-day-old seedlings grown in Fe-sufficient media were transferred to Fe-sufficient or Fe-deficient media for 4 days. Roots were sampled and used for RNA extraction. The expression of *OsIRO2* (**a**), *OsIRO3* (**b**), *OsNAAT1* (**c**), *OsYSL15* (**d**), *OsTOM1* (**e**), *OsYSL2* (**f**), *OsNAS1* (**g**), *OsNAS2* (**h**), and *OsNAS3* (**i**) was determined by quantitative real-time RT-PCR. *OsActin1* was used as the internal standard. Data are given as the means ± SD of three biological replicates. All data were compared with the WT. Significant differences from the mutant and WT are indicated by * *p* < 0.05, ** *p* < 0.01, as determined by two-tailed Student’s *t* test.

To further evaluate the role of OsIRO3 in maintaining Fe homeostasis, we examined the expression of Fe homeostasis genes in roots of *iro3* mutant and WT plants under both Fe-sufficient and Fe-deficient conditions. We selected several representative Fe homeostasis genes for analysis, including genes encoding transcriptional factors OsIRO2 and OsIRO3, and genes involved in Strategy II Fe-uptake pathways in rice [3,21,29]. Most of these genes showed a similar expression pattern between the mutant and WT plants. Specifically, the expression of all evaluated Fe homeostasis genes was significantly upregulated in the *iro3* mutants compared with that of the WT under Fe-sufficient conditions, while there was no difference in expression between WT and mutant plants under Fe-deficient conditions (Figure 5). Among the analyzed genes, *OsNAS3* expression was higher in the *iro3* mutants than that of WT plants under Fe-deficient conditions, which is different from other Fe homeostasis genes (Figure 5i). These results suggest that OsIRO3 negatively regulated Fe homeostasis genes under Fe-sufficient conditions, but negatively regulated *OsNAS3* under both Fe-sufficient and Fe-deficient conditions.

### 2.5. OsIRO3 Directly Binds to the Promoter of OsNAS3

**Figure 6:**
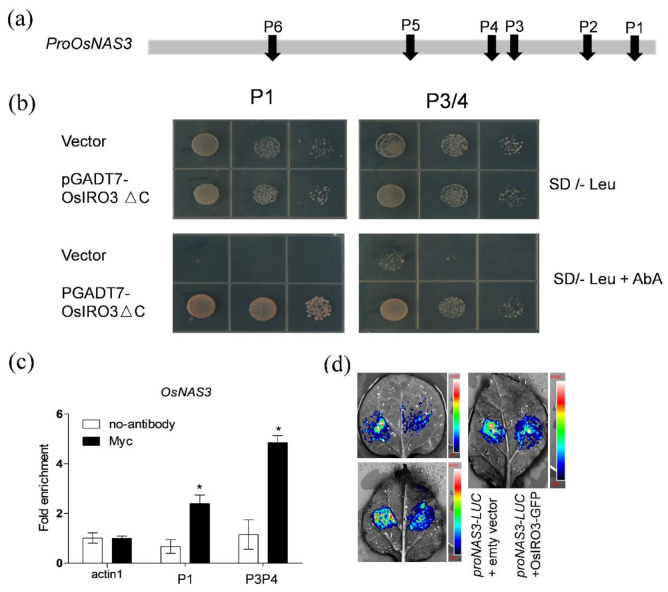
OsIRO3 binds to the promoters of *OsNAS3*. (**a**) E-boxes in the promoter. The bar indicates the position of the E-box in the 1 kb sequence from the translation start site of *OsNAS3*. (**b**) Yeast-one-hybrid assays. The P1 and P3/4 sequence indicated in (**a**) were used as bait and OsIRO3 as prey. The representative growth status of yeast cells is shown on synthetic dextrose medium agar plates without Leu (SD/-Leu) with or without aureobasidin A (AbA). The AbA resistance was activated by prey proteins that specifically interact with the bait sequence. (**c**) ChIP-qPCR analyses of the DNA binding ratio of OsIRO3 to the promoters of *OsNAS3*. qPCR was used to quantify enrichment of the indicated promoters and a fragment of the *OsActin1* promoter containing an E-box motif was used as a negative control. The DNA binding ratio indicates the targeted DNA fragment levels relative to the *OsActin1* promoter fragment. Data are given as the means ± SD of three biological replicates. Significant differences from control (no-antibody) are indicated by * *p* < 0.05, as determined by two-tailed Student’s *t* test. (**d**) OsIRO3 repressed the activity of the *OsNAS3* promoter in transient expression assays. The three pictures are representative of three experiments, respectively. The left side of tabaco leaves were co-expressed *ProOsNAS3*-LUC and empty vector, the right side of tabaco leaves were co-expressed *ProOsNAS3*-LUC and OsIRO3-GFP. In the color scale, “Red” represents a high LUC signal, while the color “Blue” represents the lowest LUC signal.

The expression pattern of *OsNAS3* suggested that it may be directly regulated by OsIRO3. To confirm this, we analyzed the 1 kb sequences upstream of the *OsNAS3* translation start sites, and identified six E-box motifs (P1–P6) that could be recognized by the bHLH transcription factor [34] (Figure 6a). Because the fragment containing P2/5/6 has self-activation in yeast one-hybrid assays, we only checked whether OsIRO3 binds to the fragment containing P1 or P3/4. Then, using a yeast one-hybrid assay, we found that OsIRO3 can bind to the fragment containing P1 or P3/4 in the *OsNAS3* promoter (Figure 6b). To further confirm the potential association of OsIRO3 with the promoters of *OsNAS3*, we performed chromatin immunoprecipitation (ChIP)-qPCR experiments (Figure 6c). Our ChIP-qPCR assays using OsIRO3-Myc plants revealed an enrichment of OsIRO3-Myc recombinant protein on the E-box motifs (P1 and P3/4) in the *OsNAS3* promoter (Figure 6c). Then, to verify the regulation effect of *OsNAS3* by OsIRO3, we analyzed the expression of pro*OsNAS3*-LUC reporter in *Nicotiana benthamiana* leaves under Pro35S:OsIRO3-GFP effector and empty vector, respectively (Figure 6d). The expression of OsIRO3-GFP in tabaco leaves was confirmed by examining the GFP signal through confocal observation (Figure S4). The Pro35S:OsIRO3-GFP effector dramatically decreased the luciferase (LUC) signal of the pro*OsNAS3*-LUC reporter (Figure 6d). Collectively, OsIRO3 appears to directly bind to the E-box of the *OsNAS3* promoter and negatively regulates its expression.

### 2.6. Nicotianamine (NA) Analysis in WT and iro3 Mutants

**Figure 7:**
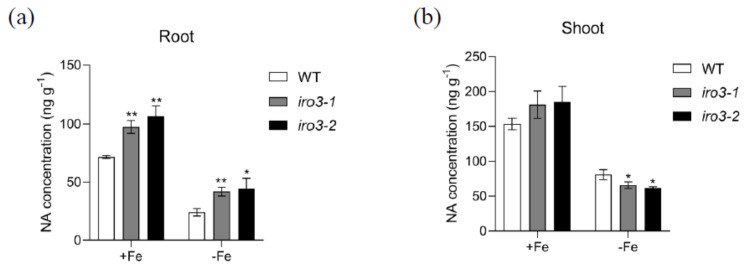
Nicotianamine (NA) concentration in WT and *iro3* mutants. (**a**) Root NA concentration of WT and *iro3* mutants under +Fe and −Fe conditions. (**b**) Shoot NA concentration of WT and *iro3* mutants under +Fe and –Fe conditions. Two-week-old seedlings were transferred to Fe-deficient conditions (−Fe) and Fe-sufficient conditions (+Fe) for 4 d. Root and shoot samples were harvested and NA concentrations were measured according to the method described in the Materials and Methods section. Data are given as the means ± SD of three biological replicates. All data were compared with the WT. Significant differences from the wild type are indicated by * *p* < 0.05, ** *p* < 0.01, as determined by two-tailed Student’s *t* test.

Given that the expression of an NA synthase–encoding gene, *OsNAS3,* was directly regulated by OsIRO3, we measured the NA concentration in WT and *iro3* mutants under both Fe-sufficient and Fe-deficient conditions. In both conditions, *iro3* mutants accumulated significantly higher levels of NA compared with WT plants (Figure 7a). This finding is consistent with the higher expression level of *OsNAS3* in the roots of *iro3* mutants compared with that of WT plants (Figure 5i). By contrast, under Fe-sufficient conditions, *iro3* mutants accumulated higher but not significantly different levels of NA compared with that of WT plants, while the NA level was lower in the *iro3* mutants compared with that of WT plants under Fe-deficient conditions (Figure 7b). These data suggest that OsIRO3 regulates Fe transport by affecting NA biosynthesis in rice.

## 3. Discussion

In plants, a number of bHLH transcription factors have been reported to be involved in modulating Fe homeostasis [35]. Among them, OsIRO3 in rice and PYE in *Arabidopsis* are negative regulators of iron homeostasis. However, OsIRO3 has been reported as a negative regulator in response to Fe deficiency only based on the functional analysis of *OsIRO3* overexpression lines [29]. In this study, by using knockout mutants of *OsIRO3,* we demonstrated that OsIRO3 is essential for the rice response to Fe deficiency and modulates the NA level by directly regulating *OsNAS3* expression.

Fe deficiency can induce some classical responses in plants, including chlorosis of new leaves, inhibition of growth development, and upregulation of Fe homeostasis genes [3,36,37]. We evaluated the role of OsIRO3 through the generation of the loss-of-function mutants of *OsIRO3* (Figures S1 and S2). Rather than exhibiting the typical Fe deficient symptom (chlorosis), the *iro3* mutants presented new leaves with severe necrosis (Figure 2a,b), with decreased shoot height and root and shoot biomass, and defective root development compared with those of WT plants (Figure 2 and Figure 3). Although both *iro3* mutants and *OsIRO3-*overexpressing lines are sensitive to Fe deficiency, *iro3* mutants contained significantly higher concentrations of Fe in the shoots under Fe-deficient conditions instead of the decreased Fe concentration observed in *OsIRO3-*overexpressing lines (Figure 4) [29]. This phenomenon also happened in the *pye* mutants [16]. Furthermore, Fe distribution in the shoots and young leaves of *iro3* mutants were not obviously different compared with those of WT plants (Figures S5 and S6). Therefore, it is hard to explain the phenotype in the mutants under Fe-deficient conditions by Fe uptake and translocation alone. PYE can directly bind to the promoters of *NAS4*, *FRO3,* and *ZIF1,* which are important for Fe distribution in plant tissues, cells, and subcellular compartments [16]. Proper Fe distribution within plant cells and subcellular compartments and Fe bioavailability are essential for normal growth and development [3,16]. We consider that hypersensitivity of *iro3* mutants to Fe deficiency could be due to disruption of Fe bioavailability and distribution homeostasis at the subcellular level. NA is an Fe chelator important for intercellular Fe homeostasis. In addition, NA is important for Fe bioavailability in plants [38,39]. In fact, we found that OsIRO3 repressed *OsNAS3* expression by directly binding to the E-box of its promoter (Figure 6). OsNAS3 is an enzyme involved in the biosynthesis of NA. Although it had been reported that PYE could directly and negatively regulate *NAS4* expression, the NA concentrations were not shown [16]. Here, we examined the NA concentration in WT and *iro3* mutants (Figure 7). The accumulation of NA in *iro3* mutants further supports the hypothesis that OsNAS3-dependent NA biosynthesis is an important cause of unbalance in Fe homeostasis in cells. Because Fe can exist in both the ferric (Fe^3+^) and the ferrous (Fe^2+^) forms in cells and can serve as an essential cofactor for components in the electron transport chain, impairment of Fe homeostasis can easily trigger the formation of harmful reactive oxygen species [1,40]. The phenomenon of new leaves of *iro3* mutants under Fe-deficient conditions was similar to the programmed cell death (PCD) induced by ROS bursts [1,40]. In our study, we demonstrated that *iro3* mutants accumulated more O_2_^−^ and H_2_O_2_ content compared to that of WT plants (Figure S3). Thus, the necrosis that appeared in the mutants may be caused by ROS bursts as a result of impaired intercellular Fe homeostasis. In addition, as graminaceous plants, NA in rice can be further used for generation of dexomugineic acid (DMA), which is essential for Fe uptake, transport, and distribution, but was not present in *Arabidopsis*. So, the potential change to the DMA concentration in *iro3* mutants is different from that of the *pye* mutants. Other uncharacterized target genes of OsIRO3 could also contribute to the hypersensitivity to Fe deficiency of *iro3* mutants. Thus, potential target genes should be investigated in the future. In addition, *OsIRO2*, *OsNAAT1*, *OsYSL15*, *OsTOM1*, *OsYSL12*, *OsNAS1,* and *OsNAS2* are important Fe homeostasis genes for Fe uptake, transport, and regulation [3,10,11,26,38,41]. The transcript abundance of these genes was significantly higher in the *iro3* mutants compared with that of the WT plants. However, unlike *OsNAS3*, the expression of these Fe homeostasis genes was not affected in the *iro3* mutants under Fe-deficient conditions (Figure 5). These data indicate that the expression of these typical Fe homeostasis genes was negatively regulated by OsIRO3 in an indirect manner. Recent studies have revealed that several subfamilies of bHLH transcription factors work together to regulate Fe homeostasis by forming homo- or heterodimers to regulate genes for Fe uptake and metabolism [14,18,22,27,35]. In *Arabidopsis*, bHLH34/104/105/115 facilitate Fe homeostasis by directly regulating the expression of *bHLH38/39/100/101* (the homologues of *OsIRO2*) and *PYE* (the homologue of *OsIRO3*) [16,17,18,19,20,25]. Furthermore, bHLH34/104/105/115 can form homo-or heterodimers [19,20]. In addition, it has been reported that PYE can form heterodimers with bHLH105 (ILR3) as a negative regulator complex of some Fe-related genes, such as *NAS4, FER,* and *NEET* [42]. This finding suggests that the interaction between Fe-related bHLH transcription factors is critical for regulating the expression of Fe homeostasis genes, like bHLH105. However, whether OsPRI1, OsPRI2, and OsPRI3 can form homo- or heterodimers or form heterodimers with OsIRO3 is unknown. Therefore, more work should be done to analyze the regulatory relationship with Fe deficiency related to bHLH proteins in rice.

Above all, OsIRO3 negatively modulates Fe homeostasis; this conserved regulatory mechanism of PYE in *Arabidopsis* and OsIRO3 in rice was further confirmed by function analysis of *iro3* mutants. Furthermore, we demonstrate *OsNAS3* is a direct target gene of OsIRO3 and is negatively regulated by OsIRO3. The hypersensitivity to Fe deficiency of *iro3* mutants and *pye* mutants indicated that the negative roles of OsIRO3 in rice and PYE in *Arabidopsis* under Fe-deficient conditions are very important.

## 4. Materials and Methods

### 4.1. Plant Materials and Growth Conditions

Wild-type (WT) rice (*Oryza sativa* cv Nipponbare) and two *iro3* mutants created by CRISPR/Cas9 [41] were used. For hydroponic experiments, plants were growth in a greenhouse at 25–30 °C. Rice seeds were soaked in water at 37 °C for 2 d. Germinated seeds were then transferred to a net floating on 0.5 mM CaCl_2_ solution. After 4 d, seedlings were transferred into half-strength Kimura B solution. The nutrient solution contained the macronutrients (NH_4_)_2_SO_4_ (0.18 mM), MgSO_4_·7H_2_O (0.27 mM), KNO_3_ (0.09 mM), Ca(NO_3_)_2_·4H_2_O (0.18 mM), and KH_2_PO_4_ (0.09 mM) and the micronutrients MnCl_2_·4H_2_O (0.50 μM), H_3_BO_3_ (3.00 μM), (NH_4_)_6_Mo_7_O_24_·4H_2_O (1.00 μM), ZnSO_4_·7H_2_O (0.40 μM), CuSO_4_·5H_2_O (0.20 μM), and Fe(III)-EDTA (50.00 μM). The pH was adjusted to 5.5, and the nutrient solution was renewed every 2 d. All experiments were repeated at least three times with three replicates each, and representative results of one experiment are shown.

### 4.2. Plasmid Construction for Plant Transformation

For generating *iro3-1* and *iro3-2* CRISPR/Cas9 mutants, two 20-bp target sequences, 5′- GCCATGGTGCCGTCGGAGAG -3′ and 5′-GCTGCCGACAAGCTCGTCCA -3′ at the first exon of *OsIRO3,* were used to design a gRNA spacer and fused to the U3 promoter at the *Bsa*I site of pRGEB31 (Addgene, Watertown, MA, USA) as described [43]. Homozygous *iro3* mutants were identified by sequencing. The coding sequence of *OsIRO3* without a stop codon was amplified and fused in frame to the 5′ terminus of Myc in the plasmid of pGWB617 to generate the vector 35S-*OsIRO3*-Myc. These constructs were introduced into the *Agrobacterium strain* EHA105. Callus was induced from mature embryos of rice cultivar Nipponbare for *Agrobacterium*-mediated rice transformation [44].

### 4.3. Phenotypic Analysis of the OsIRO3 Knockout Lines

Two-week-old seedlings of WT and two *iro3* mutants were grown in nutrient solution with 50 μM Fe (III)-EDTA or without Fe. After 8 d, root and shoot biomass and length were measured. Total root length, root surface area, and total root tip number were detected by a WinRHIZO root analysis instrument [45].

### 4.4. Fe Concentration Analysis

To compare Fe concentration in roots and shoots of WT and mutants, 14-day-old seedlings of WT, *iro3-1,* and *iro3-2* were transferred into nutrient solution containing 0 or 50 μM Fe (III)-EDTA and grown for 8 d. Roots and shoots were sampled and dried at 80 °C. After 3 d, samples were digested with HNO_3_/HClO_4_ (87:13 *v*/*v*) at 100 °C for 1 h, 120 °C for 1 h, 140 °C for 1 h, 160 °C for 1 h, and 180 °C for 1 h. After dissolving samples in 2% HNO_3_, the concentrations of Fe were determined by ICP-MS (Perkin-Elmer NexION 300X, Waltham, MA, USA).

### 4.5. Real-Time PCR Analysis

To investigate the expression pattern of *OsIRO3* in response to Fe deficiency, 2-week-old seedlings were exposed to solution without Fe for 1 week. Roots, shoots, and stems were sampled and subjected to RNA extraction. To further examine the expression pattern of different organs at different growth stages, different organs from plants grown in a paddy field were taken for RNA extraction.

For expression analysis of genes related to Fe homeostasis, WT and the *iro3* mutants were grown in a nutrient solution with or without Fe for 4 d, roots were sampled and subjected to RNA extraction. Total RNA was extracted by using an RNA extraction kit (TaKaRa, Dalian, China). A cDNA Synthesis Kit (TaKaRa, Dalian, China) was used to synthesize first-strand cDNA. Quantitative RT-PCR was performed using the SYBR Green Supermix system on a Mastercycler ep realplex machine (Eppendorf, Germany). *OsActin1* was used as the internal standard. The primers used for quantitative real-time PCR are listed in Table S1.

### 4.6. Yeast-One-Hybrid Assay

For yeast one hybrid assay, the fragments containing P1 and P3/4 of the *OsNAS3* promoter were inserted into the pAbAi vector and the coding sequences of *OsIRO3* were cloned into the pGADT7 prey vector. The plasmids were co-transferred into the Y1H Gold yeast strain according to the Matchmaker Gold Yeast One-Hybrid Library Screening System (Clontech). Yeast strains can grow well on the synthetic dextrose medium without Leu supplemented with 200 ng/mL aureobasidin A (AbA), which indicates interaction between prey protein and the bait sequence.

### 4.7. Transient Expression Assays in Tobacco

To investigate the regulatory role of OsIRO3 on *OsNAS3* expression in tobacco, we constructed an effector vector Pro35S:*IRO3-*GFP and a reporter vector Pro*OsNAS*3:LUC. *Agrobacterium tumefaciens* strain EHA105 was used. The corresponding constructs were co-transiently expressed in young leaves of tobacco by an *Agrobacterium*-mediated infiltration method as described previously [20]. Tobacco leaves were infected by agrobacterial cells containing plasmids by an infiltration buffer (10 mM MgCl_2_, 0.2 mM acetosyringone, and 10 mM MES, pH 5.6).

### 4.8. ChIP-qPCR Assay

Pro35S:*OsIRO3-*Myc transgenic lines were used for the ChIP assays according to previously described protocols [46,47]. Roots of Pro35S:*OsIRO3-*Myc transgenic plants were cross-linked with 1% (*v*/*v*) formaldehyde under vacuum for 30 min, and Gly was added to a final concentration of 0.125 mol L^−1^ to quench the cross-linking. Then, samples were immediately ground in liquid nitrogen for nuclei isolation, and the chromatin solution was then sonicated to shear the DNA into fragments of 100~1000 bp. Protein chromatin DNA complexes were isolated by Myc Antibody (Santa). To check OsIRO3-DNA binding efficiency, qPCR was performed according to the procedure described previously. *pOsACTIN1* was used as the endogenous control.

### 4.9. Measurement of Nicotianamine (NA) Concentration

To determine the NA concentrations in WT and the *iro3* mutants, 2-week-old seedlings were exposed to solution with or without Fe. After 4 d, root and shoot were sampled and stored at −20 °C before measurement. Then, 100 mg of samples were ground in liquid nitrogen and extracted with 500 μL of water at 80 °C for 30 min, followed by 10 min centrifugation (18,000× *g*). The supernatant solution was transferred to centrifuge tubes with filters (Amicon Ultra) and further centrifuged for 10 to 30 min at 18,000× *g*. The NA concentration in the supernatant solution was determined using a UPLC interconnected with a LTQ Orbitrap XL mass spectrometer (Thermo Scientific, Waltham, MA, USA) as described previously [48].

### 4.10. Bioimaging of Fe by μ-XRF

The new leaves were sampled from 14-day-old seedlings of both WT and *iro3* mutants (*iro3-2*) after Fe deficiency treatment for 4 d. The new leaves were put on the 4% agarose for µ-XRF analysis. The high-resolution distribution analysis of Fe in the new leaves was analyzed using a micro X-ray Fluorescence (μ-XRF) Spectrometer (M4 Tornado) [49]. The Pixel size was 4 μm and Pixel time was 4 ms.

### 4.11. Measurement of ROS Level in New Leaves

Nitro blue tetrazolium (NBT) staining was used to detect O_2_^−^, and H_2_O_2_ was detected by 3,3′–diaminobenzidine (DAB) staining. The new leaves were sampled from 14-day-old seedlings of both WT and *iro3* mutants after Fe deficiency treatment for 4 d. Then, the samples were cut into small pieces and were submerged in NBT solution (6 mM NBT prepared in 10 mM of sodium citrate, pH = 6) and DAB solution (1 mg/mL DAB solution, pH = 3.8) in a Petri dish (35 mm) using tweezers. Samples were then incubated at room temperature for 8 h under light [50]. After incubation, samples were discolored in 95% boiling ethanol until the chlorophyll was removed completely. The cleared leaves were then photographed.

### 4.12. Statistical Analyses

Statistical analysis was performed using SPSS ver. 20.0 for all the obtained data. For pairwise comparisons of WT and *iro3* mutants, data were analyzed using one-way ANOVA followed by two-tailed Student’s *t* test.

## Supplementary Materials

The following are available online at https://www.mdpi.com/2223-7747/9/9/1095/s1, Figure S1: Gene editing of *OsIRO3*. Figure S2. The predicted truncated proteins in the *iro3* mutants. Figure S3. Loss of OsIRO3 function results in increased of ROS level. Figure S4. Picture of OsIRO3-GFP signal in tabaco leaves. Figure S5. Fe distribution in shoot of *iro3* mutants. Figure S6. Metal distribution in new leaves of WT and *iro3-2*. Table S1: Primers used in this study.
