# Supplementary material for: OsIRO3 Plays an Essential Role in Iron Deficiency Responses and Regulates Iron Homeostasis in Rice
Source: Plants (Basel). 2020 Aug 25;9(9):1095. doi: 10.3390/plants9091095 (PMC7570094; doi:10.3390/plants9091095)
Supplement: Supplementary file 1 [file plants-09-01095-s001.zip › plants-903599 - supplementary figures for XML.pdf]

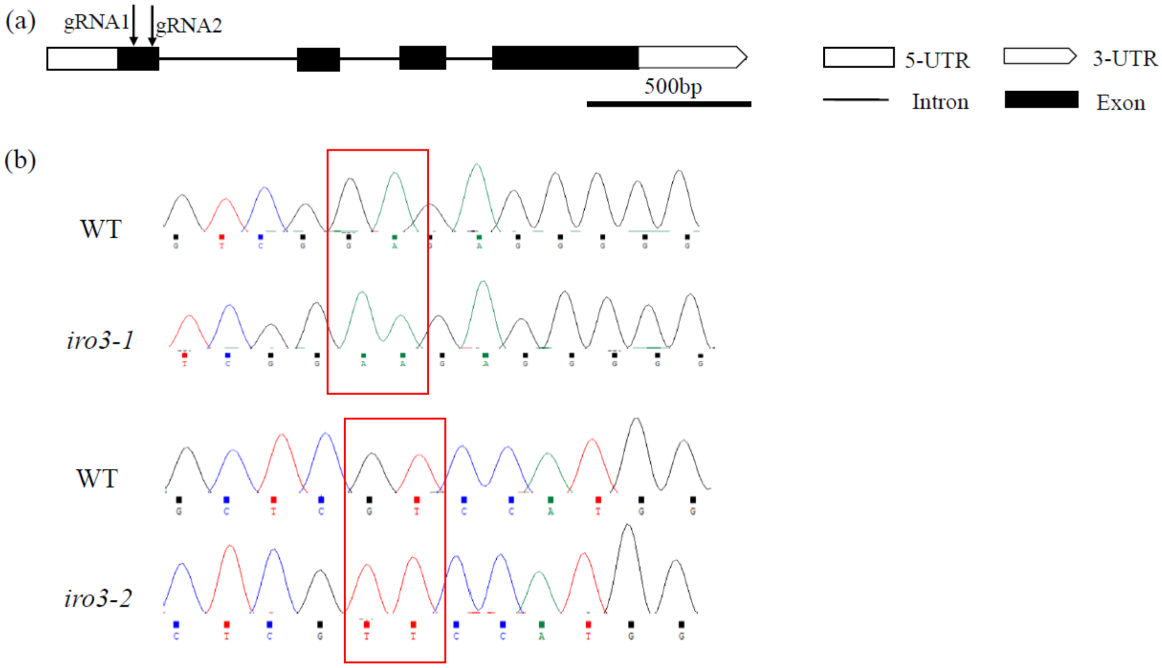

Figure S1. Gene editing of *OsIRO3*. (**a**) Schematic diagram of the *OsIRO3* gene structure and the gRNA sites. (**b**) The results of sequence from *iro3-1* and *iro3-2*. The *iro3-1* mutant contains an insertion of 'A'; the *iro3-2* mutant contains an insertion of 'T'.

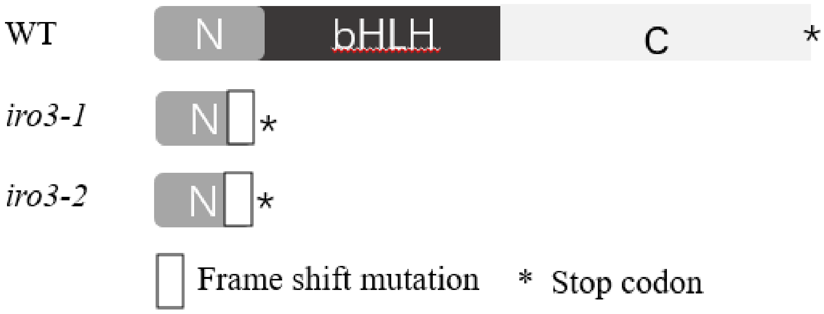

Figure S2. The predicted truncated proteins in the WT and *iro3* mutants. WT, wild type; *iro3-1* and *iro3-2* represent two independent knockout mutants of *OsIRO3*, the *iro3-1* mutant contains an insertion of 'A'; the *iro3-2* mutant contains an insertion of 'T'. * represents stop codon.

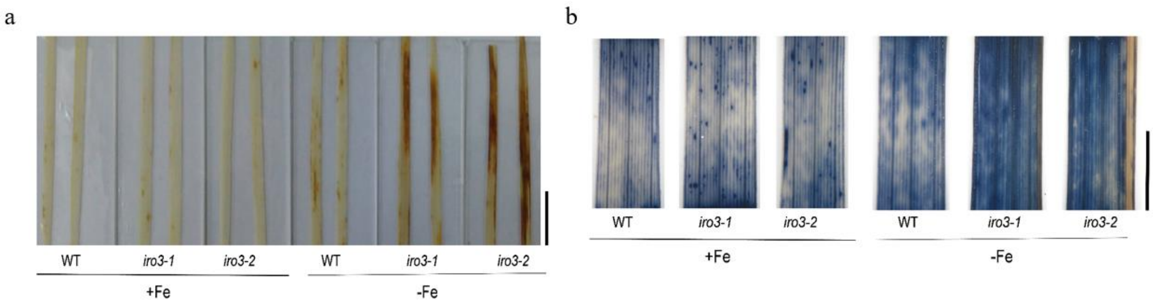

Figure S3. Loss of OsIRO3 function results in increased of ROS level. Fourteen-day-old seedlings of WT, *iro3-1*, and *iro3-2* responded to iron deficiency for 4 d and new leaves were sampled for detected ROS level. (**a**) 3, 30-diaminobenzidine (DAB) staining, bar = 1 cm; (**b**) Nitrotetrazolium Blue chloride (NBT) staining, bar = 0.5 cm. WT, wild type; *iro3-1* and *iro3-2* represent two independent *OsIRO3*

knockout mutants, the *iro3-1* mutant contains an insertion of 'A'; the *iro3-2* mutant contains an insertion of 'T'.

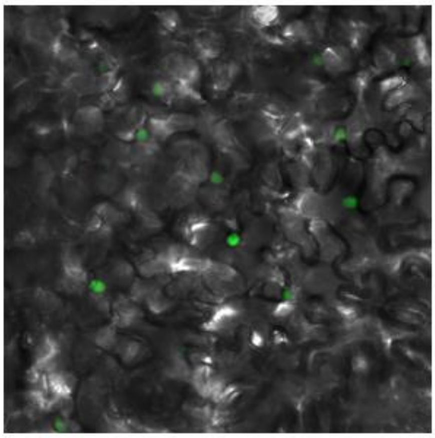

Figure S4. Picture of OsIRO3-GFP signal in tabaco leaves. Confocal images tabaco epidermis cells expressing OsIRO3-GFP.

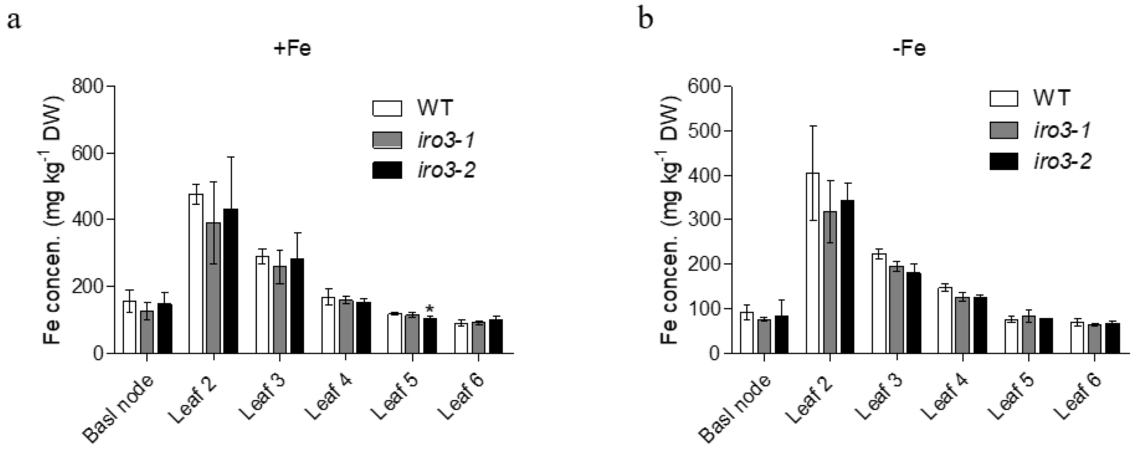

Figure S5. Fe distribution in shoot of *iro3* mutants. Eighteen-day-old seedlings of WT, *iro3-1*, and *iro3-2* were shifted to nutrient solution containing 0 and 2 μM Fe^{2+} and grown for 14 d. Individual leaf blades (leaf blades 2 to 6) and base node (1 cm distance from root and shoot junction) were sampled for Fe analysis. Fe content in individual leaf blades and base node under Fe sufficient conditions (**a**) and Fe-deficient conditions (**b**). Asterisks indicate significant differences of WT and *iro3* mutants based on two-tailed Student's *t* test (* $p < 0.05$).

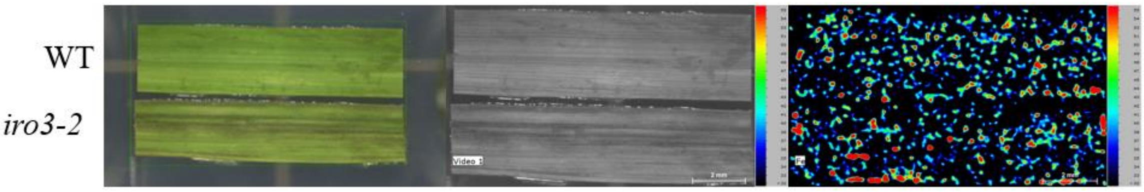

Figure S6. Metal distribution in new leaves of WT and *iro3-2*. Fourteen-day-old seedlings of WT and *iro3-2* were shifted to nutrient solution with or without Fe for 6 d. Plants new leaf were used for detected Fe distribution. WT, wild type; *iro3-2* represents *OsIRO3* knockout mutant, the *iro3-2* mutant contains an insertion of 'T'.
